# Transition From Open Trigger Finger Release to Micro-incision Trigger Finger Release Under Real-Time Ultrasound Guidance: A Single-Surgeon Experience With 133 Consecutive Cases

**DOI:** 10.7759/cureus.110816

**Published:** 2026-06-14

**Authors:** Diane C Riley, Brendan E Hines

**Affiliations:** 1 Orthopaedics, Alice Peck Day Memorial Hospital, Dartmouth Health, Lebanon, USA; 2 Orthopaedics, Geisel School of Medicine at Dartmouth, Hanover, USA

**Keywords:** elective hand surgery, open trigger finger, percutaneous trigger finger release, post-operative management, sonosurgery, trigger finger release, ultrasound, ultrasound-guided, ultrasound guided trigger finger release

## Abstract

Purpose

The primary objective of this review was to report the results of trigger finger release (TFR) under real-time ultrasound (US) guidance in a real-world population, including patients with concomitant hand issues and other comorbidities.

Method

A retrospective review of the corresponding author’s case log for 2023 was conducted to identify all patients who underwent an index, middle, ring, or little finger micro-incision TFR from February through September of that year under real-time US guidance (ultrasound TFR (uTFR)). The preoperative demographics collected included triggering severity, comorbidities, age, and sex. The postoperative course included the number and type of postoperative visits required and notation of intraoperative or postoperative complications.

Results

Overall, 209 TFRs were performed on 116 consecutive patients using uTFR. There were no revision surgeries, nerve injuries, or infections. One digit required an intraoperative conversion to an open TFR (oTFR) with flexor digitorum superficialis (FDS) slip resection for persistent locking. Notably, 114 of the patients (98%) had at least one medical comorbidity or concomitant hand procedure at the time of uTFR. Furthermore, 113 patients (97%) were eligible for a streamlined follow-up process, and 104 (92%) elected to have only a telephone or registered nurse (RN) visit for follow-up after surgery. Seven patients (6%) had a post-procedure steroid injection on the operative digit, and 18 (16%) attended at least one occupational therapy (OT) visit.

Conclusion

Microincision uTFR appears to be safe for the index, middle, ring, and little fingers regardless of comorbidities. Multiple digits can be released at the same time, even under local anesthesia. The technique allows for a streamlined process for most patients and for the practice.

## Introduction

Approximately 300,000 trigger finger releases (TFRs) are performed each year in the United States. Most are done openly. The success rate of open TFR (oTFR) is reported to be 90% to 100% [1-3]. Recent publications, however, have shown that the postoperative course can be prolonged and complicated, especially from the patient’s perspective [4-9]. The revision surgical rates for oTFR at one and five years were recently found to be higher than previously reported (1.81% and 3.85% at one and five years, respectively, compared with a range of 0.4% to 2.4% in previous reports) [10-12].

Since 1958, surgeons have worked to develop a percutaneous TFR procedure to avoid scar tissue formation, stiffness, and bowstringing [13]. Multiple studies have reported that percutaneous releases are safe and effective. Nevertheless, cadaveric and clinical studies have shown that non-image-guided percutaneous techniques have led to incomplete pulley release, tendon scarring, and nerve injury, especially for border digits of the index and little fingers [14-29]. To address this concern, Jou and Chern utilized an ultrasound (US)-guided percutaneous release with a 21-gauge needle and a hook knife and achieved a 97% success rate [24].

US technology has advanced significantly since 2006. The advancements in imaging have led to increased interest and reports of improved outcomes for not only percutaneous TFR but also carpal tunnel releases [24,25,30-39]. The corresponding author converted to real-time, US-guided carpal tunnel release from mini open release on February 1, 2022, using a commercially available device (UltraGuideCTR, Sonex Health, Eagan, MN, USA). This conversion eliminated the need for sutures and thus involved changing the postoperative course from multiple in-person follow-ups, including the next clinic day after the procedure and at least two additional visits for suture removal and motion check, to a telehealth visit or phone call and further follow-up only if needed. The change in technique created a more streamlined process in both the clinic and the operating room by eliminating the need for clinic visits and reducing both instrumentation in the operating room and turnover time.

The UltraGuideCTR technique has proved to be highly reliable, with no need to convert to an open carpal tunnel release in more than 1,000 cases. Thus, when the US Federal Drug Administration (FDA) approved the UltraGuideTFR device (Sonex Health, Eagan, MN, USA) for use at the corresponding author’s institution on February 28, 2023, she decided to determine whether the same streamlined process adopted for carpal tunnel releases would be effective for trigger finger patients. From that date forward, the corresponding author offered microincision (3 mm) TFR under real-time US guidance with the UltraGuideTFR device to all patients requiring release of an index, middle, ring, or little finger.

Prospective trials have shown that percutaneous TFR led to a faster recovery with a better cosmetic result than open surgery. Systematic reviews further concluded that US-guided TFR (uTFR) had a 97% success rate [30,35,39]. Most series, however, have limited the study populations by excluding patients with comorbidities of diabetes mellitus (DM), osteoarthritis (OA), Dupuytren’s, inflammatory arthritis, multiple trigger digits, or, sometimes, prior trigger fingers [24,25,30-35,37,38]. In addition, studies have generally excluded patients who were being treated with other hand procedures at the same time they received TFR using US guidance [24,25,30-38,40]. To address these knowledge gaps, this study presents a retrospective chart review of all patients who underwent TFR of an index, middle, ring, or little finger in a single surgeon’s practice using the UltraGuideTFR. No patient was excluded based on the number of triggering digits released at the time of surgery, treatment with concomitant procedures at the time of surgery, or comorbidities.

The aim of this study was to report the results of TFR under real-time US guidance in a real-world population, including patients with concomitant hand issues and other comorbidities. The primary objective was to determine the safety and effectiveness of uTFR to resolve triggering in the index, middle, ring, and little fingers, with a secondary objective to determine if the technique allowed for a change in postoperative management.

## Materials and methods

A retrospective review of the corresponding author’s case log for 2023 was conducted to identify all patients who had undergone an index, middle, ring, or little finger TFR from February 2023 through September 2023. In total, 133 consecutive cases were performed on 117 patients, all utilizing the commercially available UltraGuideTFR device specifically designed for a micro-incision technique under real-time US guidance. All patients were older than 18 years and able to give consent for office and surgical treatment. No patient was considered part of a vulnerable population. The inclusion criteria were patients aged 18 or older with triggering of the index, middle, ring, or little finger that warranted surgical release during the date range of the study. No patient was excluded based on the number of triggering digits, concomitant procedures at the time of surgery, or comorbidities. Trigger thumbs were excluded from the micro-incision technique because the UltraGuideTFR device is not approved for thumbs.

The surgeries were performed in an operating room. Irrespective of the type of anesthesia administered, all patients received local field blocks of 1% lidocaine with 1:100,000 epinephrine mixed with bicarbonate of soda at a 9:1 ratio. After infiltration of the skin and the sheath, a 3 mm incision was made under direct US guidance to allow the introduction of the UltraGuideTFR device. The position of the device was confirmed to be beneath the A1 pulley in the short axis and advanced to the A1-A2 junction. The cutting blade was activated, and the pulley was released in a retrograde fashion. The patients were asked to actively flex and extend their fingers to confirm the cessation of snapping or locking. The technique is demonstrated in Video 1.

**Video 1:** Trigger finger release under real-time ultrasound guidance.

In cases in which there was no triggering or locking, Mastisol® and Steri-strips® were applied, followed by a compressive dressing. One finger demonstrated persistent triggering or locking despite confirmation of the A1 pulley release on US. This digit was converted to an open release under direct 3.5 loupe magnification. All open trigger finger incisions were closed with interrupted 5.0 nylon sutures in addition to the compressive wrap. The possibility of needing to convert to an open trigger finger was included in all consents and preoperative consultations.

The operative notes were reviewed for the patients’ age, sex, type of anesthetic, specific digit, and number of digits released as well as any concomitant hand surgery procedures. Early complications were considered, including the need to convert to an open procedure for persistent triggering or locking, bleeding, nerve injury, tendon rupture, or infection. Late complications were also considered, including return to an operating room for a procedure related to the TFR, loss of motion, progression of Dupuytren’s warranting intervention, and recurrence of triggering.

The preoperative notes were reviewed for age, sex, medical comorbidities, severity of trigger fingers, prior TFR, and the length and complexity of recovery after any prior release. Trigger finger severity was graded based on the Quinnell grades [41,42]. The micro-incision UltraGuideTFR technique does not require sutures, so patients were offered the option of either in-person or telephone follow-up. Telephone notes, clinic notes, and occupational therapy (OT) notes were reviewed for two years postoperatively to identify the need for follow-up in person, referral to OT, and/or the need for an injection or additional surgery.

**Figure 1:**
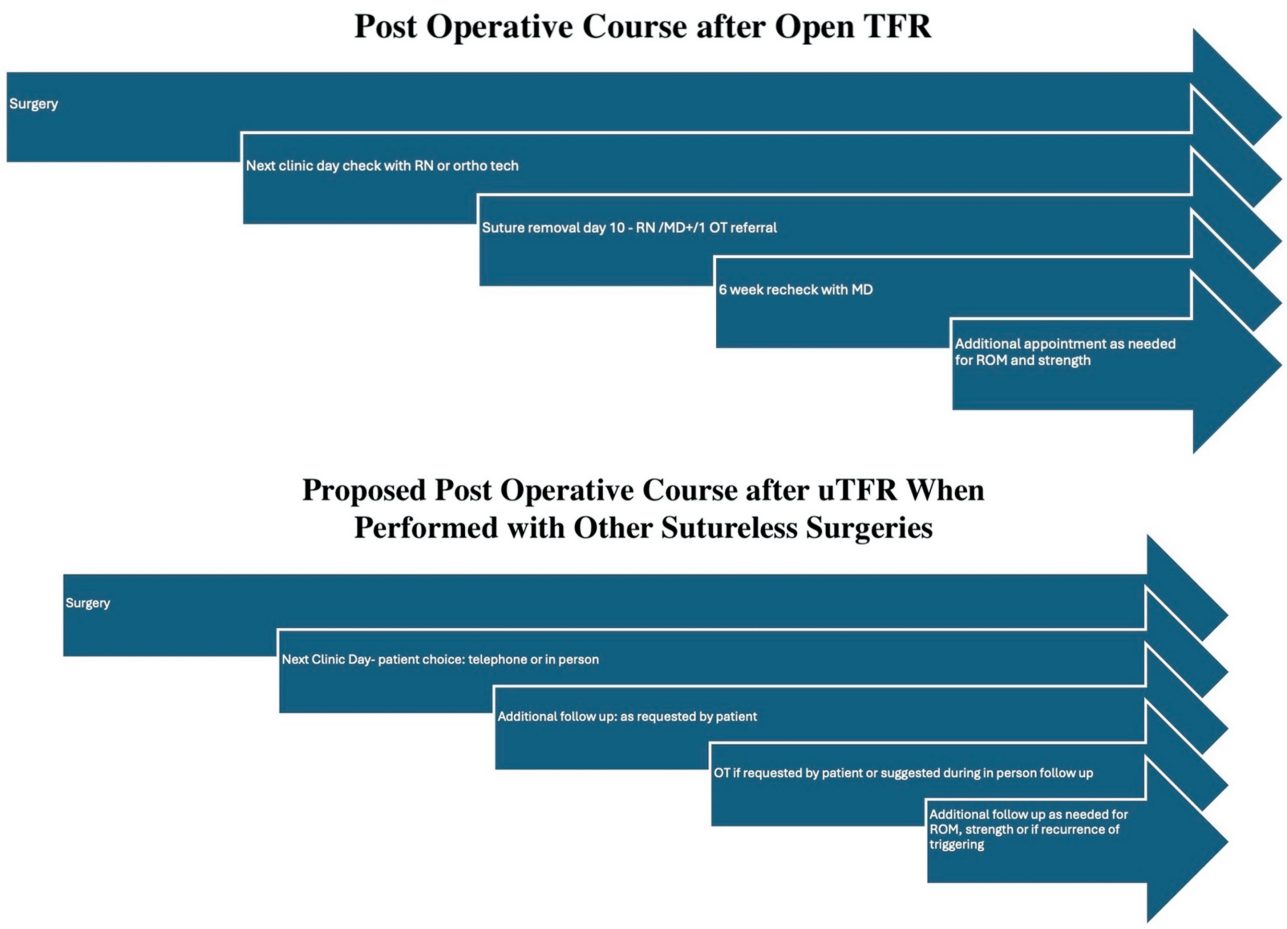
Postoperative course of oTFR compared with uTFR. uTFR: ultrasound trigger finger release; oTFR: open trigger finger release; TFR: trigger finger release; RN: registered nurse; MD: medical doctor; OT: occupational therapy; ROM: range of motion

All in-person visits, whether by a registered nurse (RN), hand therapist, physician assistant, or physician, involved evaluating the patient for infection, range of motion, and resolution of snapping or locking. The resolution of symptoms for patients who selected telephone follow-up was considered confirmed when documented in a phone note or in an in-person visit when a patient returned for a different medical problem. Figure 1 summarizes the change in postoperative management proposed for the US-guided release compared with oTFR.

## Results

In total, 211 uTFRs were performed on 117 consecutive patients in the study period. One patient was lost to follow-up, leaving 209 trigger fingers in 116 patients. Overall, 16 patients had staged surgeries (32 cases). Of the cohort, 80 patients were female and 37 were male. The patients ranged in age from 39 to 92 years. In total, 116 of the patients (99%) had continuity of care in our electronic medical record (Epic). Overall, 90 cases (68%) were performed under local anesthesia and 42 (32%) under monitored anesthesia care. Restoration of motion and resolution of snapping and locking at the time of surgery were achieved in 208 digits (99.5%) in 116 patients. One patient required conversion to an oTFR because of persistent triggering and locking after the UltraGuideTFR release. That patient had two other digits released with the micro-incision UltraGuideTFR at the same time. The digit that required conversion to open was found to have had a full release of the pulley by the UltraGuideTFR but had a significantly frayed ulnar slip of the flexor digitorum superficialis (FDS) tendon, which was treated with partial FDS resection. The patient had experienced triggering in this digit for more than two years, had received more than three steroid injections, and had preoperative loss of motion. This patient required OT and a steroid injection postoperatively for exacerbation of Dupuytren’s in the digit that required FDS resection, but not in the two other digits released with the UltraGuideTFR at the same time. In total, 43 other digits (21%) were Quinnell grade 4, but none required conversion to open. No other intraoperative complications were observed; therefore, no patient demonstrated snapping, locking, intraoperative bleeding, nerve injury, tendon rupture, or infection.

The patients’ demographics are summarized in Table 1. Overall, 18 patients (16%) were diabetic, 22 (19%) had thyroid disease, 72 (62%) had OA, 25 (22%) had autoimmune diseases, and 41 (35%) had prior open trigger fingers. In total, 35 patients (30%) had prior oTFRs of the index, middle, ring, or little fingers; of these, 19 (54%) had documentation of post-procedure OT, seven (20%) did not require OT, and no information on the need for OT was available for nine (26%). A single-digit TFR was performed in 23 cases (17%), whereas multiple digits and/or additional surgical procedures or injections were performed in 109 cases (83%).

**Table 1:** Patient demographics. # denotes number of fingers released; ? denotes no Quinnell grade recorded. M: male; F: female; ANA, antinuclear antibody; PMR: polymyalgia rheumatica; GCA: giant cell arteritis; PsA: psoriatic arthritis; RA: rheumatoid arthritis; DM: diabetes mellitus; OA: osteoarthritis; oTFR: open trigger finger release; Ds: disease; CTR: carpal tunnel release; CUTR; cubital tunnel release; TFR: trigger finger release

Patient Demographics		
Age	30-39	1
	40-49	3
	50-59	28
	60-69	36
	70-79	44
	80+	5
Sex	M	37
	F	80
Comorbidities	Autoimmune	-
	ANA (+) not specified	3
	Crohn’s	1
	Crystalline	5
	PMR/GCA	3
	PsA	4
	Post viral	4
	RA	5
	DM	18
	OA	72
	Prior oTFR	41
	Thyroid ds	22
Anesthesia	Mac	42
	Local	90
Side	Left	56
	Right	71
	Bilateral	5
# UTFR	1	73
	2	46
	3	8
	4	5
Digit	Index	28
	Middle	91
	Ring	65
	Little	25
Quinnell	?	3
	2	88
	3	65
	4	53
Concomitant		88
CTR		42
CUTR		1
Cyst aspiration		1
Dequervains		4
Injection		26
Thumb TFR		14

**Figure 2:**
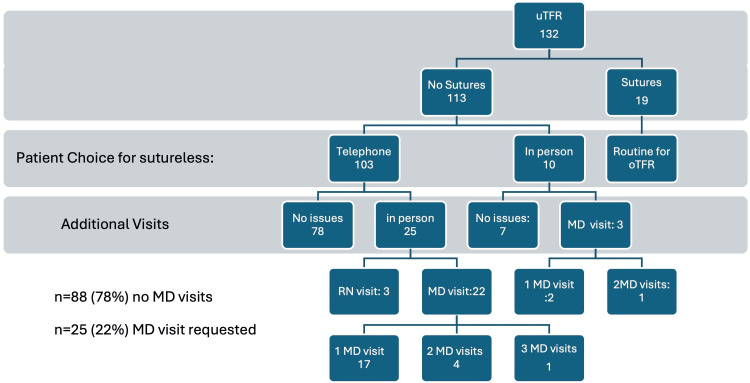
Postoperative course for uTFR only patients. uTFR: ultrasound trigger finger release; RN: registered nurse; MD: medical doctor

In 113 cases (97%), the patients were offered the choice of in-person follow-up or telephone follow-up. Overall, 78 (76%) of these cases had telephone follow-up only. In total, 85 patients (75%) who selected telephone-only follow-up or an in-person RN visit did not require any further care. While there were no reported issues in any telephone note, the notes did not specifically state whether the resolution of snapping and locking occurred in 22 cases (19%). Of these, 11 of the 22 patients (50%) had prior TFRs. Overall, 88 of the 113 patients offered a choice of follow-up (78%) did not request follow-up with a medical doctor (MD), while 25 (22%) requested an in-person visit. Of the 25 patients (22%) who had at least one in-person visit with an MD, seven (28%) had successful postoperative steroid injections for spot weld (six patients) at the incision or stiffness (one patient), and nine (36%) were referred to OT. Of these nine patients, three had locked trigger fingers, and four underwent staged procedures. Figure 2 summarizes the postoperative uTFR visits for the patients who did not have sutures.

**Figure 3:**
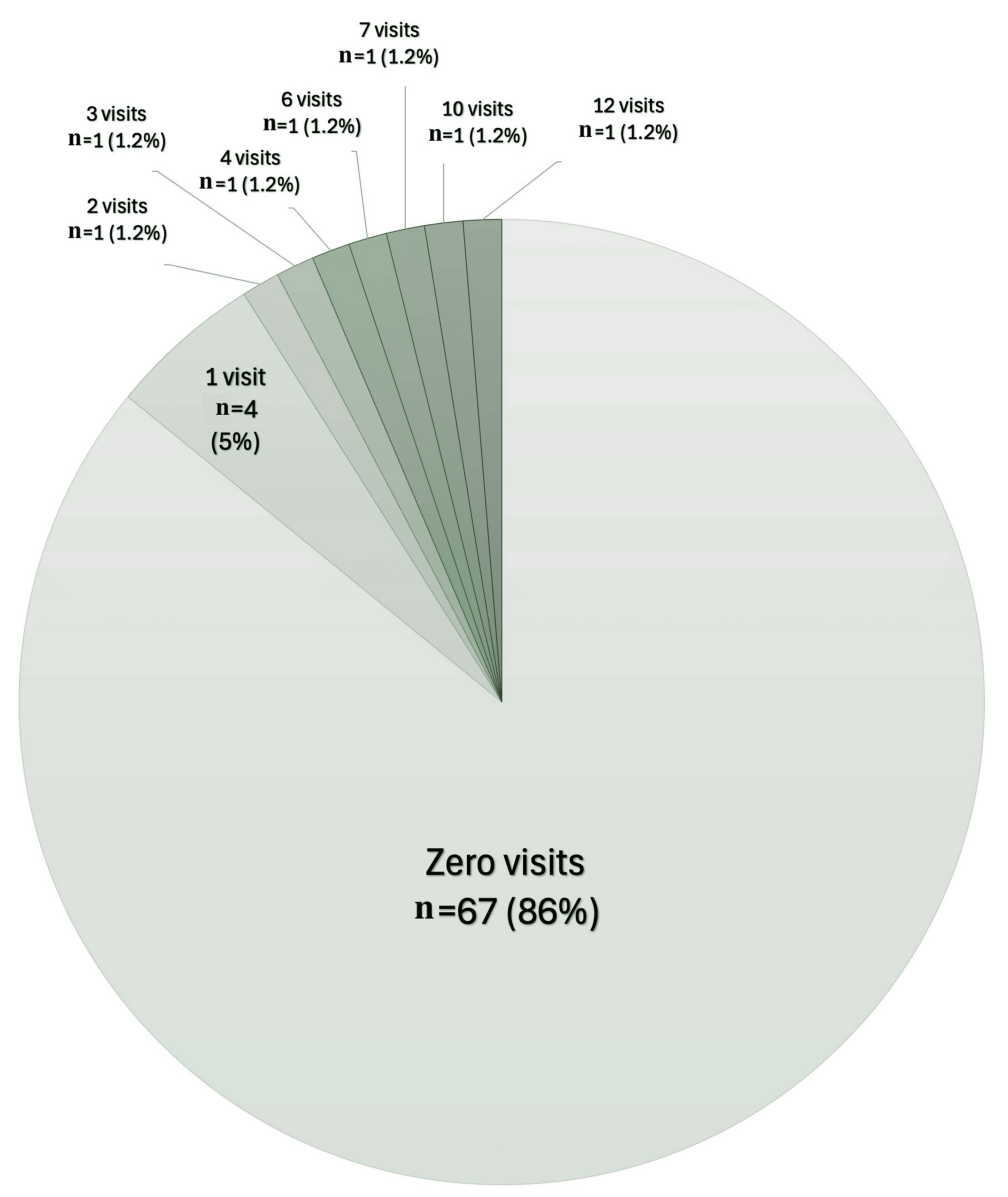
OT visits for uTFR-only patients. OT: occupational therapy; uTFR: ultrasound trigger finger release; n: number of patients

Ten percent of the patients with trigger finger only were referred for OT. Figure 3 summarizes the number of OT visits per patient after uTFR in the patients whose sole reason for OT was the uTFR procedure.

**Figure 4:**
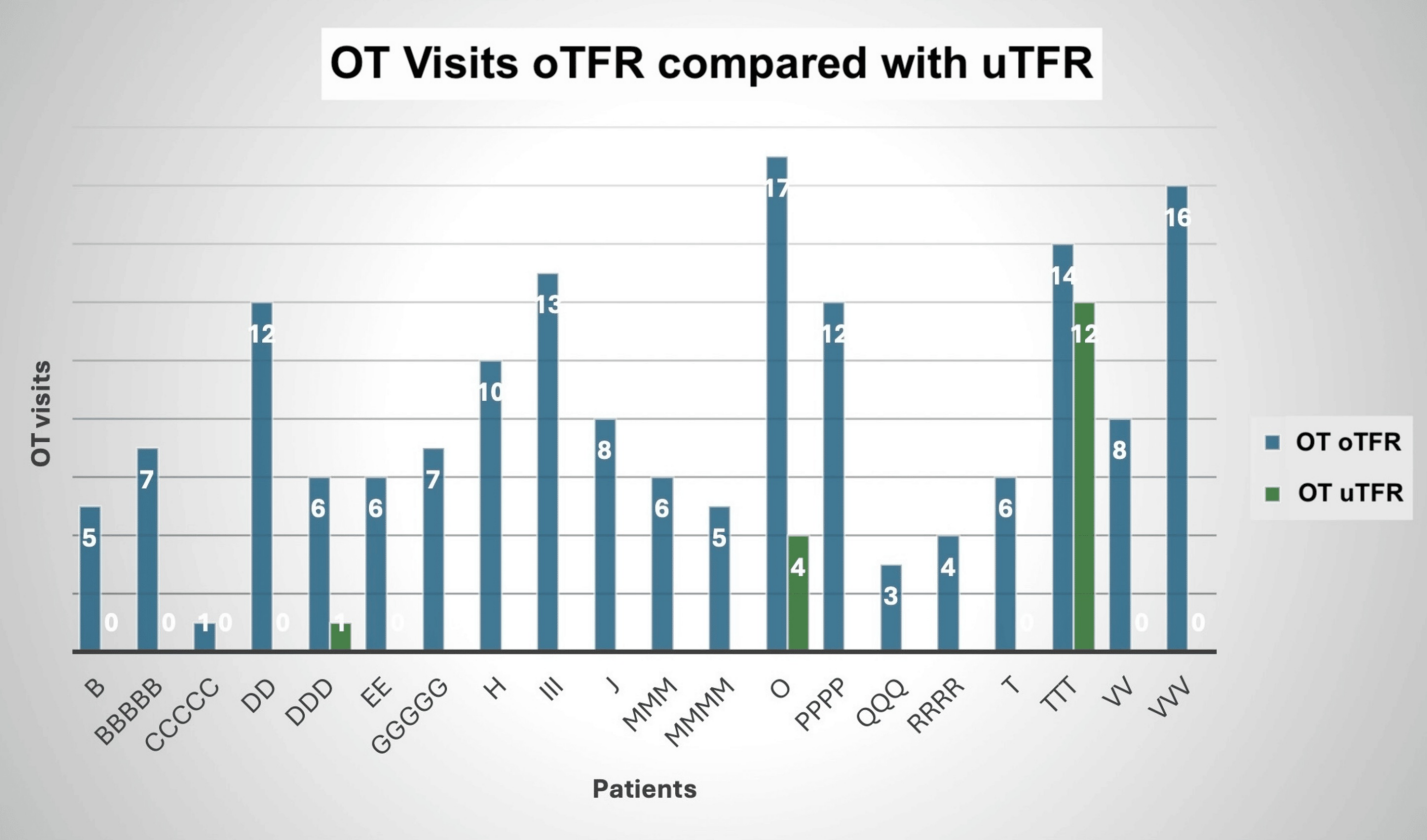
Comparison of OT visits after oTFR or uTFR. The y-axis represents the deidentification codes for individual patients. OT: occupational therapy; oTFR: open trigger finger release; uTFR: ultrasound trigger finger release

All patients who had previously undergone an oTFR required fewer visits after their uTFR procedures (Figure 4). 

## Discussion

Micro-incision UltraGuideTFR under real-time US guidance is a safe and effective procedure. None of the patients in the series had recurrence of triggering, nerve injury, infection, or need for additional surgery. There was one intraoperative conversion from the UltraGuideTFR technique to an oTFR for a Quinnell grade 4 trigger finger that was snapping after UltraGuideTFR. The A1 pulley was fully released, but the patient had a shredded slip of the FDS requiring resection. Overall, the need for FDS slip resection was lower than that reported for open trigger fingers by Favre and Kinnen (12%) [43], Kwan et al. (1.2%) [44], or Fisher et al. (5.8%) [45]. The patient in our study had multiple triggering digits, and the digit that required resection had been locking and contracted for more than one year. Fisher et al. found that all of these factors increased the odds ratios of requiring FDS resection [45].

The technique safely and effectively resolved triggering irrespective of the digit released or the patients’ comorbidities. Single or multiple digits can be released at the same time under local anesthesia or monitored anesthesia. The results of this series correlate with the findings of prior studies reporting on the effectiveness of US-guided percutaneous TFR [24,25,30-39,46]. A major difference from earlier studies is that the present series was larger and included comorbidities and concomitant hand surgeries. The technique was equally effective in patients with inflammatory arthritis, including RA, whereas an earlier study did not recommend TFR in patients with RA [8]. The patients in this series with RA were receiving treatment from a rheumatologist, and none had joint subluxation or angulation at the metacarpal phalangeal joint at the time of the TFR. Thus, inflammatory arthritis may not be a contraindication to pulley release for patients who are receiving adequate treatment for it.

The resolution of triggering was reaffirmed in the postoperative period in all but 14 of the cases that received telephone follow-up only. Six of these patients had recovered successfully from prior TFRs (three uTFR cases and three oTFR cases). Ninety percent of the 17 patients who underwent sutureless procedures required no care beyond a telephone or RN visit. This finding represents a significant change in the postoperative course for the practice. Nineteen patients in this series (16%) developed new trigger digits, and all of them chose uTFR over oTFR. Consequently, uTFR with the option for telephone-only follow-up has become the corresponding author’s standard practice. The change in postoperative management from in-person follow-up at three days, removal of sutures at 10 days, and MD follow-up at six weeks to telephone-only follow-up at three days saved patients, support staff members, and the physician 301, 103, and 85 visits, respectively. These savings correlated with reductions in in-person visits for the patients, support staff members, and the physician of 89%, 96%, and 75%, respectively.

Seven patients received postoperative steroid injections in the uTFR digit, in two cases for stiffness, in one case for Dupuytren’s, and in four cases for a spot weld at the incision. Lee et al. reported that 1 in 20 patients who underwent US-guided release with a hook knife required postoperative steroid injections for tenosynovitis at six weeks, though they excluded patients with OA, RA, or combined diseases in the same hand [25]. While the post-procedure injection rate in the present series was the same as in the study by Lee et al., the patients requiring injection were more diverse: all had Dupuytren’s, four had multiple locking digits, four had OA, two had psoriatic arthritis, and one had carpal tunnel release. Three other studies have reported the results of uTFR with steroid injections, but the injections in these studies were performed at the time of the procedure rather than postoperatively [47-49]. Bruijnzeel et al. reported that 14 (0.02%) of the patients in their study required steroid injections after oTFR: three (18%) for persistent triggering, one for recurrent triggering (7%), and 10 (72%) for slow recovery. The steroid injections were very effective in resolving stiffness and/or concerns about Dupuytren’s in all seven patients requiring post-procedure injections in the present study.

The need for OT after TFR varies greatly. Bruijnzeel et al. reported a 2% referral rate to OT [6]. Pompeu et al. reported a considerably higher rate, and the management of the patients in their study following oTFR included general hand therapy [7]. Chung reported the results of 16 trigger digits released under US guidance with a Kemis H1 knife; all of the patients in their study received both physical therapy (PT) and OT [50]. Their patients averaged 2.7 sessions of PT and 2.9 sessions of OT (two patients had one PT visit and one OT visit, and the remainder ranged from two to nine OT visits and two to six PT visits). These researchers also found a greater need for OT and PT in patients with inflammatory arthritis, multiple digit releases, or joint deformities.

In the present study, 17 patients (13%) who received sutureless procedures attended OT: five had concomitant carpal tunnel release, five had multiple trigger digits released at the same time, and seven had only one session of OT. Of the 10 patients requiring more than one session of OT, three had insulin-dependent DM, eight had Dupuytren’s, four had OA, and three underwent carpal tunnel release. Regardless of comorbidities or the number of procedures performed during the same surgery, all of the patients in this series required less OT than they received for open procedures.

This series was limited because it was a retrospective single-surgeon case series. Every effort was made to cross-check the accuracy of the number of visits for follow-up and/or OT after the use of both the open and the UltraguideTFR techniques. However, some of the patients’ oTFRs were performed at outside hospitals, some were performed more than a decade previously, and the notes from the outside institutions and/or conversion to the medical record were missing. Thus, while the uTFRs were performed by a single surgeon in a seven-month time frame, the oTFRs were performed by a variety of surgeons over several decades with less consistency in the information on comorbidities, concomitant procedures, and follow-up needs. As a result, our results may underrepresent the amount of OT that patients received after their oTFR procedures. Patients who received telephone follow-up in this series may have experienced stiffness that was not evident at the time of the calls but did not feel the need for in-person follow-up. The telephone follow-ups were performed by multiple nurses and orthopedic technologists. Thus, minor complications may have been underreported in their documentation. While all pertinent notes in the electronic medical records were reviewed, the patients may have visited an institution outside the network, in which case their notes would not have been incorporated into Epic and could contribute to potential underreporting of postoperative complications.

A second limitation of the present study is the utilization of OT visits as a proxy for the length of postoperative recovery. Some patients may have had a more prolonged recovery, but did not desire to attend OT or be seen in the clinic.

Another limitation of the study is the grading system used to assess trigger fingers. The Quinnell grading system is based on palpation and the observation of snapping and locking [41]. The Green and Froimson classifications are derivatives of the Quinnell system that do not involve classification of tendon integrity or tendinopathy [51,52]. Abdul Nasir et al. conducted a prospective study of 136 open trigger fingers and found that 70% of cases had some flexor tendon degeneration, though, interestingly, the degeneration correlated not with the Green classification grade, but, rather, with the number of preoperative steroid injections [53]. This percentage is similar to the sonographic findings of 63% by Guerini et al. [54]. It is well-recognized that the duration of symptoms, the number of preoperative steroid injections, flexion contracture of the proximal interphalangeal joint, a history of multiple trigger fingers, Dupuytren’s, DM, carpal tunnel syndrome, De Quervain’s, and partial or tendon fraying are risk factors for prolonged postoperative symptoms [4,5,12]. Being male, diabetic, and/or older than 65 years and having carpal tunnel, hypertension, or ischemic heart disease are risk factors for needing revision surgery [10].

Pompeu et al. pointed out the inadequacy of the present grading systems for addressing this more extensive pathoanatomy, rightly observing that US is a dynamic means to assess changes to the pulley, tendons, and joints [7]. US is well-suited to visualizing the subsurface structures, but further studies are needed to correlate the present understanding of the grading systems for the severity of trigger fingers with the US findings.

## Conclusions

We found micro-incision uTFR to be a safe and effective method for the index, middle, ring, and little fingers, even in the presence of medical comorbidities. Multiple digits can be released at the same time under local anesthesia. There were no revisions, nerve injuries, or infections among the patients in our study. Because uTFR is a sutureless technique, in-person follow-up was not needed or requested by 70% of the patients. This change saved 301 visits for patients, 226 visits for the RN and members of the support staff, and 85 visits for the physician. Thus, this more streamlined follow-up process eliminated unnecessary visits for both the patients and the practice.

## References

[1] Lange-Riess D, Schuh R, Hönle W, Schuh A (2009). Long-term results of surgical release of trigger finger and trigger thumb in adults. Arch Orthop Trauma Surg.

[2] Lim MH, Lim KK, Rasheed MZ, Narayanan S, Beng-Hoi Tan A (2007). Outcome of open trigger digit release. J Hand Surg Eur.

[3] Turowski GA, Zdankiewicz PD, Thomson JG (1997). The results of surgical treatment of trigger finger. J Hand Surg Am.

[4] Baek JH, Chung DW, Lee JH (2019). Factors causing prolonged postoperative symptoms despite absence of complications after A1 pulley release for trigger finger. J Hand Surg Am.

[5] Blazar PE, Zhang D, Bryant JK, Benavent KA, Yeung CM, Earp BE (2024). Patient-perceived outcomes of recovery after trigger digit release. J Hand Surg Am.

[6] Bruijnzeel H, Neuhaus V, Fostvedt S, Jupiter JB, Mudgal CS, Ring DC (2012). Adverse events of open A1 pulley release for idiopathic trigger finger. J Hand Surg Am.

[7] Pompeu Y, Aristega Almeida B, Kunze K, Altman E, Fufa DT (2021). Current concepts in the management of advanced trigger finger: a critical analysis review. JBJS Rev.

[8] Ryzewicz M, Wolf JM (2006). Trigger digits: principles, management, and complications. J Hand Surg Am.

[9] Will R, Lubahn J (2010). Complications of open trigger finger release. J Hand Surg Am.

[10] Weaver DJ, Lewis J, Abdelfadeel W, Strelzow JA, Wolf JM (2025). Revision A1 pulley release: an analysis of risk factors using a national database. J Hand Surg Am.

[11] Koopman JE, Zweedijk BE, Hundepool CA (2022). Prevalence and risk factors for postoperative complications following open A1 pulley release for a trigger finger or thumb. J Hand Surg Am.

[12] Everding NG, Bishop GB, Belyea CM, Soong MC (2015). Risk factors for complications of open trigger finger release. Hand (N Y).

[13] Lorthioir J (1958). Surgical treatment of trigger-finger by a subcutaneous method. J Bone Joint Surg Am.

[14] Eastwood DM, Gupta KJ, Johnson DP (1992). Percutaneous release of the trigger finger: an office procedure. J Hand Surg Am.

[15] Abdoli A, Asadian M, Banadaky SH, Sarram R (2021). A cadaveric assessment of percutaneous trigger finger release with 15° stab knife: its effectiveness and complications. J Orthop Surg Res.

[16] Aksoy A, Sir E (2019). Complications of percutaneous release of the trigger finger. Cureus.

[17] Bain GI, Turnbull J, Charles MN, Roth JH, Richards RS (1995). Percutaneous A1 pulley release: a cadaveric study. J Hand Surg Am.

[18] Bamroongshawgasame T (2010). A comparison of open and percutaneous pulley release in trigger digits. J Med Assoc Thai.

[19] Calleja H, Tanchuling A, Alagar D, Tapia C, Macalalad A (2010). Anatomic outcome of percutaneous release among patients with trigger finger. J Hand Surg Am.

[20] Dunn MJ, Pess GM (1999). Percutaneous trigger finger release: a comparison of a new push knife and a 19-gauge needle in a cadaveric model. J Hand Surg Am.

[21] Fu YC, Huang PJ, Tien YC, Lu YM, Fu HH, Lin GT (2006). Revision of incompletely released trigger fingers by percutaneous release: results and complications. J Hand Surg Am.

[22] Habbu R, Putnam MD, Adams JE (2012). Percutaneous release of the A1 pulley: a cadaver study. J Hand Surg Am.

[23] Hoang D, Lin AC, Essilfie A (2016). Evaluation of percutaneous first annular pulley release: efficacy and complications in a perfused cadaveric study. J Hand Surg Am.

[24] Jou IM, Chern TC (2006). Sonographically assisted percutaneous release of the a1 pulley: a new surgical technique for treating trigger digit. J Hand Surg Br.

[25] Lee SH, Choi YC, Kang HJ (2018). Comparative study of ultrasonography-guided percutaneous A1 pulley release versus blinded percutaneous A1 pulley release. J Orthop Surg (Hong Kong).

[26] Pope DF, Wolfe SW (1995). Safety and efficacy of percutaneous trigger finger release. J Hand Surg Am.

[27] Ragoowansi R, Acornley A, Khoo CT (2005). Percutaneous trigger finger release: the 'lift-cut' technique. Br J Plast Surg.

[28] Smith J, Rizzo M, Lai JK (2010). Sonographically guided percutaneous first annular pulley release: cadaveric safety study of needle and knife techniques. J Ultrasound Med.

[29] Tanaka J, Muraji M, Negoro H, Yamashita H, Nakano T, Nakano K (1990). Subcutaneous release of trigger thumb and fingers in 210 fingers. J Hand Surg Br.

[30] Amro S, Kashbour M, Shaaban Abdelgalil M, Qafesha RM, Eldeeb H (2024). Efficacy of ultrasound-guided tendon release for trigger finger compared with open surgery: a systematic review and meta-analysis. J Ultrasound Med.

[31] Chopin C, Le Guillou A, Salmon JH, Lellouche H, Richette P, Maillet J (2022). Treatment of trigger finger by ultrasound-guided needle release of A1 pulley: a series of 105 cases. Jt Bone Spine.

[32] Colberg RE, Pantuosco J, Fleisig G, Drogosz M (2020). Ultrasound-guided microinvasive trigger finger release technique combined with three Tests to confirm a complete release. Am J Phys Med Rehabil.

[33] Rojo-Manaute JM, Rodríguez-Maruri G, Capa-Grasa A, Chana-Rodríguez F, Soto Mdel V, Martín JV (2012). Sonographically guided intrasheath percutaneous release of the first annular pulley for trigger digits, part 1: clinical efficacy and safety. J Ultrasound Med.

[34] Rodríguez-Maruri G, Rojo-Manaute JM, Capa-Grasa A, Chana Rodríguez F, Cerezo López E, Vaquero Martín J (2023). Ultrasound-guided A1 pulley release versus classic open surgery for trigger digit: a randomized clinical trial. J Ultrasound Med.

[35] Nakagawa H, Redmond T, Colberg R (2023). Ultrasound-guided A1 pulley release: a systematic review. J Ultrasound Med.

[36] Nikolaou VS, Malahias MA, Kaseta MK, Sourlas I, Babis GC (2017). Comparative clinical study of ultrasound-guided A1 pulley release vs open surgical intervention in the treatment of trigger finger. World J Orthop.

[37] Pan M, Sheng S, Fan Z (2019). Ultrasound-guided percutaneous release of A1 pulley by using a needle knife: a prospective study of 41 cases. Front Pharmacol.

[38] Yavari M, Modaresi SM, Hassanpour SE, Moosavizadeh SM, Tabrizi A (2023). Clinical study between percutaneous ultrasound-guided release and open classic surgery in treating multiple trigger fingers. Adv Biomed Res.

[39] Zhao JG, Kan SL, Zhao L (2014). Percutaneous first annular pulley release for trigger digits: a systematic review and meta-analysis of current evidence. J Hand Surg Am.

[40] Atthakomol P, Manosroi W, Sathiraleela K (2023). Prognostic factors related to recurrence of trigger finger after open surgical release in adults. J Plast Reconstr Aesthet Surg.

[41] Gil JA, Hresko AM, Weiss AC (2020). Current concepts in the management of trigger finger in adults. J Am Acad Orthop Surg.

[42] Quinnell RC (1980). Conservative management of trigger finger. Practitioner.

[43] Favre Y, Kinnen L (2012). Resection of the flexor digitorum superficialis for trigger finger with proximal interphalangeal joint positional contracture. J Hand Surg Am.

[44] Kwan SA, Sherman MB, Fletcher D, Matzon JL (2025). Risk factors for requiring ulnar superficialis slip resection during trigger finger release. J Hand Surg Am.

[45] Fisher MM, Allen AD, Jeffs AD (2025). A comparison of patient characteristics and outcomes between patients receiving flexor digitorum superficialis slip excision or isolated A1 pulley release for trigger finger. J Hand Surg Am.

[46] Saremi H, Hakhamaneshi E, Rabiei MA (2016). Percutaneous release of trigger fingers: comparing multiple digits with single digit involvement. Arch Bone Jt Surg.

[47] Jegal M, Woo SJ, Il Lee H, Shim JW, Park MJ (2019). Effects of simultaneous steroid injection after percutaneous trigger finger release: a randomized controlled trial. J Hand Surg Eur.

[48] White RZ, Sampson MJ (2021). Assessment of short-term response and review of technique of ultrasound-guided percutaneous A1 pulley release for the treatment of trigger finger. J Med Imaging Radiat Oncol.

[49] Wu YY, He FD, Chen K, Quan JR, Guo XY (2020). Comparison of the clinical effectiveness of ultrasound-guided corticosteroid injection with and without needle release of the A1 pulley in treating trigger finger. J Xray Sci Technol.

[50] Chung MMT (2025). Ultrasound-guided minimally invasive trigger finger release in a Chinese population. J Orthop Rep.

[51] Wolfe SW (2017). Green's Operative Hand Surgery.

[52] Boyer M (1999). Trigger finger. Green's Operative Hand Surgery.

[53] Abdul Nasir M, Ahmad TS, Low TH, Devarajooh C, Gunasagaran J (2023). Flexor tendon degeneration affects short-term outcomes of open trigger digit release. PLoS One.

[54] Guerini H, Pessis E, Theumann N (2008). Sonographic appearance of trigger fingers. J Ultrasound Med.

